# Normative database for separate inner retinal layers thickness using spectral domain optical coherence tomography in Caucasian population

**DOI:** 10.1371/journal.pone.0180450

**Published:** 2017-07-05

**Authors:** María Nieves-Moreno, Jose M. Martínez-de-la-Casa, Pilar Cifuentes-Canorea, Marina Sastre-Ibáñez, Enrique Santos-Bueso, Federico Sáenz-Francés, Laura Morales-Fernández, Julián García-Feijoó

**Affiliations:** Servicio de Oftalmología, Hospital Clínico San Carlos; Departamento de Oftalmología, Facultad de Medicina, Universidad Complutense de Madrid; and Instituto de Investigación Sanitaria del Hospital Clínico San Carlos, Madrid, Spain; Charite Universitatsmedizin Berlin, GERMANY

## Abstract

**Purpose:**

Develop the first normative database of the thickness of every inner retinal layer in the macular area in a healthy, Caucasian population between 18 to 87 years old, using Spectralis Optical Coherence Tomography (OCT).

**Methods:**

On this transversal, observational study, 300 patients between 18 to 87 years old and without an ophthalmological condition were recruited. Macular OCT scans were performed on all patients (Spectralis OCT, Heidelberg Engineering). An axial length measurement, and keratometry were performed using an optical biometer. The volume and thickness of the different macular sectors of the inner retinal layers (retinal nerve fiber layer (RNFL), ganglion cells layer (CGL) and inner plexiform layer (IPL)) were analyzed with the Spectralis OCT segmentation software. An eye was randomly selected for each patient.

**Results:**

297 patients (179 females and 118 males) were included in the study. The mean age was 56.07 years (range: 40.50–72). The multivariate analysis showed a positive correlation between the RNFL thickness and the axial length (p < 0.001). The mean central retinal thickness was 278.2 μm (range: 266–291), the mean central RNFL thickness was 12.61 μm (range: 11–14), the mean central CGL thickness was 17.63 μm (range: 14–21) and the mean central IPL thickness was 22.02 μm (range: 20–25). The multivariate analysis showed a negative correlation between age and CGL thickness and inner IPL thickness (p< 0.001).

**Conclusion:**

This study provides a normative database of the volume of each of the inner retinal layers on a Caucasian population.

## Introduction

Glaucoma is one of the most frequent causes of irreversible blindness in developed countries. Its pathogenesis is characterized by a progressive loss of retinal ganglion cells (RGCs) which translates into a progressive loss of the visual field, and in advanced stages can lead to blindness[1]. Given the irreversible character of the illness, detecting the glaucomatous changes in a reliable way as soon as possible is very important.

The arrival of the Optical Coherence Tomography (OCT) has made a big change in the management and diagnosis of retinal pathologies and glaucoma. The OCT is a non-invasive transpupilar imaging method that produces high-resolution, cross-sectional tomographic images of the retinal tissue that are in good correlation with retinal histology[2,3]. With spectral domain OCTs (SD-OCTs), it is possible to acquire images quicker and higher in quality than with time domain OCTs (TD-OCTs)[4], The latest version of the Spectralis SD-OCT software (Heidelberg Engineering) allows the automatic segmentation of the retinal layers individually, and this analysis has been proven recently to have an excellent repeatability and reproducibility in healthy subjects[5].

To optimize the diagnostic tests results, they are generally compared with normative databases performed with subjects not presenting the disease[6]. Currently, to the authors' knowledge, a normative database for the thickness of each layer within the inner retinal tissue in the macular area for Spectralis SD-OCT does not exist.

The purpose of this study was to establish the normal macular thickness values of the inner retinal layers in healthy Caucasian volunteers: retinal nerve fiber layer (RNFL), ganglion cell layer (GCL) and inner plexiform layer (IPL).

## Methods

This study was an observational, cross-sectional analysis of three hundred eyes of 300 normal subjects of Caucasian origin. The subjects were selected consecutively from January 2015 to February 2016, they were referred to the hospital for a routine examination or graduation. The study was approved by the Institutional Review Board of the Hospital Clínico San Carlos (Madrid, Spain) and it followed the guidelines from the Helsinki declaration.

### Participants

This study included 300 healthy Caucasian volunteers without any eye problem, all participants were >18 years old. All subjects signed an informed consent after hearing a detailed explanation about the tests that were going to be conducted on the study and its purpose. All subjects were interviewed about their ancestries and only subjects with Caucasian origin were included in this study, subjects with biracial ancestry were not included in this study. A full ophthalmic examination was performed, including an evaluation of the medical record, biomicroscopy, measurement of the intraocular pressure (IOP), eye fundus test, refraction and axial length. For each patient, one eye was randomly selected for the final analysis. Patient selection was made following the recommendations of Realini et al[6].

Every eye included in the study had a visual acuity equal or greater than 20/40, a sphere between +/-5 diopters and a cylinder between +/-3 diopters. Patients with macular abnormalities were excluded, as well as the patients with recent eye surgery (<1 year) or personal medical history of IOP >21 mmHg, glaucoma, macular or retinal diseases. Patients with personal medical history of neurological diseases or any other uncontrolled disease were also excluded.

### Optical coherence tomography

SD-OCT macular examination was performed the same day without pupil dilation in a dark room using Spectralis OCT (Heidelberg Engineering). This scan was performed on a 20x20 degree cube with 49 Raster lines separated by 120u, each containing 1046 pixels. The automatic eye tracking technology maintains fixation on the retina. Only well-centered images with a signal strength of >20db were used for analysis.

Macular and inner retinal layers thickness were reported in an Early Treatment of Diabetic Retinopathy Study macular map (ETDRS). The 1, 3 and 6 mm rings were considered for the analysis. The 1 mm ring was defined as central thickness. The intermediate and outer rings were divided into four zones designated as superior, nasal, inferior, and temporal. The numerical values recorded for each of the nine zones and the macular volume were used in the analysis.

The new Spectralis segmentation software (version 6.0c) was used to obtain individual retinal layer thickness measurement including retinal thickness, retinal nerve fiber layer (RNFL), ganglion cell layer (GCL) and inner plexiform layer (IPL). All scans were performed by the same experienced operator and no manual adjustments to retinal layer segmentation were made.

### Statistical analysis

One eye of each subject was selected randomly. Descriptive statistics were reported as mean, range and standard deviation, as well as the 1^st^, 5^th^ and 95^th^ percentiles. Normal macular thickness values were compared among the quadrants using the paired t-test. All p-values were adjusted by the Bonferroni factor. Correlation between different measurements was done using the Pearson correlation coefficients. Multivariate regression analysis was used to analyze the effects of age, gender and axial length, p < 0,05 was considered significant. All statistical analysis were performed using the SPSS package version 15 (SPSS Inc., Chicago, IL, USA)

## Results

Three hundred Caucasian subjects were examined. Three cases had artifacts in macular segmentation and had to be excluded. Two hundred and ninety-seven subjects were included in this study (179 women and 118 men). The mean age was 56.07 +/- 18.72 years (range: 40.50–72). The mean axial length was 23.44 +/- 1.14 mm (range: 22.61–24.16) The mean keratometry was 43.89 +/- 3.04 (range: 42.93–44.06). After the randomization, 150 right eyes and 147 left eyes were included. On Table 1, the characteristics of the subjects for each age group are shown.

**Table 1:** Characteristics of the subjects by age group.

				Axial Length	Keratomety
Age group	N° Subjects	Sex (males, %)	Mean (SD)		Mean (SD)
18–33	56	23 (41%)	23,93 (1,11)		42,75 (1,49)
34–50	56	19 (33%)	23,72 (1,22)		34,76 (1,46)
51–68	94	38 (40%)	23,28 (1,13)		44,08 (1,58)
69–87	91	38 (42%)	23,14 (1,03)		44,45 (1,54)

The mean retinal volume was 8.58 +/- 0.36 mm^3^ (range: 8.36–8.81), the mean RNFL volume was 0.96 +/- 0.12 mm^3^ (0.89–1.01 range), the mean ganglion cell layer volume was 1.03 +/- 0.12 mm^3^ (range: 0.95–1.11) and the mean inner plexiform volume was 0.88 +/- 0.7 mm^3^ (range: 0.84–0.93). The retina, GCL and IPL volumes were significantly higher in male subjects in comparison to female subjects (p<0.001, p = 0.002 and p = 0.025 respectively). In Tables 2–5 the volume and the macular thickness distribution are shown on the different sectors for each of the inner retinal layers.

**Table 2:** Distribution of macular values using Spectralis-SD-OCT in normal subjects.

RETINA	18–33 years				34–50 years				51–68 years				69–87 years			
		Percentile				Percentile				Percentile				Percentile		
	Mean	1st	5th	95th	Mean	1st	5th	95th	Mean	1st	5th	95th	Mean	1st	5th	95th
**Vol (mm3)**	8,74	8,18	8,28	9,16	8,71	8,07	8,17	9,28	8,59	7,08	8,17	9,13	8,41	7,40	7,72	9,02
**Central (μm)**	277	220	244	309	280	238	250	311	279	220	247	319	277	214	235	315
**Nasal inner (μm)**	351	325	243	372	349	325	328	372	344	313	322	367	337	300	306	368
**Nasal outer (μm)**	322	294	300	343	317	295	297	341	313	280	294	335	305	259	275	328
**Superior inner (μm)**	347	314	325	363	348	324	329	370	342	314	319	363	334	293	305	364
**Superior outer (μm)**	302	281	283	323	302	278	285	329	298	309	312	361	392	256	265	309
**Temporal inner (μm)**	331	299	306	352	334	309	311	361	329	302	309	349	323	279	299	352
**Temporal outer (μm)**	284	267	269	299	285	256	265	309	281	252	263	300	277	244	251	303
**Inferior inner (μm)**	345	318	322	370	344	321	325	370	339	309	318	361	332	292	301	361
**Inferior outer (μm)**	292	275	277	309	290	260	268	311	285	258	267	310	281	245	257	301

**Table 3:** Distribution of macular RNFL values using Spectralis-SD-OCT in normal subjects.

RNFL	18–33 years				34–50 years				51–68 years				69–87 years			
		Percentile				Percentile				Percentile				Percentile		
	Mean	1st	5th	95th	Mean	1st	5th	95th	Mean	1st	5th	95th	Mean	1st	5th	95th
**Vol (mm3)**	0,95	0,74	0,78	1,12	0,97	0,72	0,80	1,21	0,95	0,75	0,78	1,12	0,96	0,66	0,77	0,21
**Central (μm)**	12,8	7	9	16	12,5	8	8,90	15	12,7	6	9	16,3	12,9	6	8,60	17
**Nasal inner (μm)**	22,1	18	19	26	22,1	18	18	26	21,9	16	18	26,3	21,6	18	18	27
**Nasal outer (μm)**	53,2	38	39,9	68,3	52,9	39	40,9	65,3	51,5	37	39,8	66,3	50,3	27	36,8	96,4
**Superior inner (μm)**	24,8	19	20	30	24,9	19	20	30,1	24,2	19	20	28	24,7	19	20	32
**Superior outer (μm)**	37,7	29	29	48	39,6	22	32	53,4	37,9	29	29,8	44,5	39,7	25	26,6	52,4
**Temporal inner (μm)**	17,3	15	15,9	19,2	17,8	16	16	19	17,8	15	16	20	18,3	15	16	21
**Temporal outer (μm)**	18,7	16	16,9	21,3	19,3	17	17	22,2	19,9	16	17	22	20,7	17	18	24
**Inferior inner (μm)**	26,2	21	21,9	32,2	27,2	20	21,9	34	26,5	20	21	32,5	25,6	16	19,6	32
**Inferior outer (μm)**	41,4	30	31,2	51,6	42,4	27	33,4	52,8	41,4	30	32,8	55	41,1	26	30,6	54,4

**Table 4:** Distribution of macular GCL values using Spectralis-SD-OCT in normal subjects.

GCL	18–33 years					34–50 years				51–68 years				69–87 years			
		Percentile					Percentile				Percentile				Percentile		
	Mean	1st	5th	95th	Mean		1st	5th	95th	Mean	1st	5th	95th	Mean	1st	5th	95th
**Vol (mm3)**	1,03	0,77	0,83	1,21	1,05		0,79	0,82	1,23	1,05	0,72	0,87	1,21	0,99	0,74	0,77	1,16
**Central (μm)**	19,6	11	12	27	18,4		10	11,9	27,1	17,1	7	10	26	16,6	7	10	26
**Nasal inner (μm)**	50,7	39	40,7	59,2	51,0		31	41	60,2	49,9	37	41	59	46,9	30	36	58,4
**Nasal outer (μm)**	35,0	24	25,9	43,2	35,4		21	25,9	44,3	35,8	22	28	42	34,0	22	24	41
**Superior inner (μm)**	49,6	35	38,9	59,2	50,6		37	39,9	59,3	50,2	34	39,8	58	47,4	32	36,6	58
**Superior outer (μm)**	32,9	23	24	40,2	33,6		26	26,9	42,1	33,9	22	27	40	32,1	23	24	38
**Temporal inner (μm)**	46,5	37	37,9	56	47,4		37	39,7	54,2	46,4	36	38	55	43,1	26	32,2	53
**Temporal outer (μm)**	34,9	25	28,9	42	35,0		24	27,8	24,2	34,8	24	29,8	41,3	33,1	22	26	40,8
**Inferior inner (μm)**	49,5	37	38,7	60	50,6		34	39,9	59,2	50,2	34	40	58	47,0	33	37	58
**Inferior outer (μm)**	31,5	22	24	38,2	31,3		23	24	38,2	31,7	20	25,8	38	30,6	22	23,6	36

**Table 5:** Distribution of macular IPL values using Spectralis-SD-OCT in normal subjects.

IPL	18–33 years				34–50 years				51–68 years				69–87 years			
		Percentile				Percentile				Percentile				Percentile		
	Mean	1st	5th	95th	Mean	1st	5th	95th	Mean	1st	5th	95th	Mean	1st	5th	95th
**Vol (mm3)**	0,91	0,77	0,79	1,01	0,91	0,77	0,79	1,04	0,89	0,72	0,77	1,00	0,85	0,65	0,72	0,96
**Central (μm)**	22,6	16	17	28	22,3	16	17	27,9	22,0	15	16,8	27,9	21,7	13	16	28,8
**Nasal inner (μm)**	44,0	39	39	48	43,6	31	37,9	48,6	42,5	37	37	48	40,1	29	33,6	47
**Nasal outer (μm)**	29,5	24	24,9	34	29,1	21	23,7	34,2	28,8	22	24	33,5	27,0	19	21,6	31,4
**Superior inner (μm)**	42,2	35	37,7	46,2	24,4	36	38,7	47,5	41,1	32	36	46,3	39,0	28	31,6	45
**Superior outer (μm)**	28,7	23	24	33	28,8	24	24,9	33	28,4	22	25	33	26,8	19	22	32
**Temporal inner (μm)**	42,2	37	37	47	43,0	36	37	49,2	41,7	32	34,8	47	39,6	29	32,6	45,4
**Temporal outer (μm)**	31,9	25	27	36,3	32,3	24	27,9	37	31,8	24	27	37,3	30,8	24	26	36,4
**Inferior inner (μm)**	42,1	37	37	46,1	42,2	34	36,9	48	41,0	33	35,8	45	38,6	29	31,6	45
**Inferior outer (μm)**	27,5	22	23	32,2	26,8	22	22	31,3	26,6	20	23	32	25,6	20	21,2	29,8

**Fig 1:**
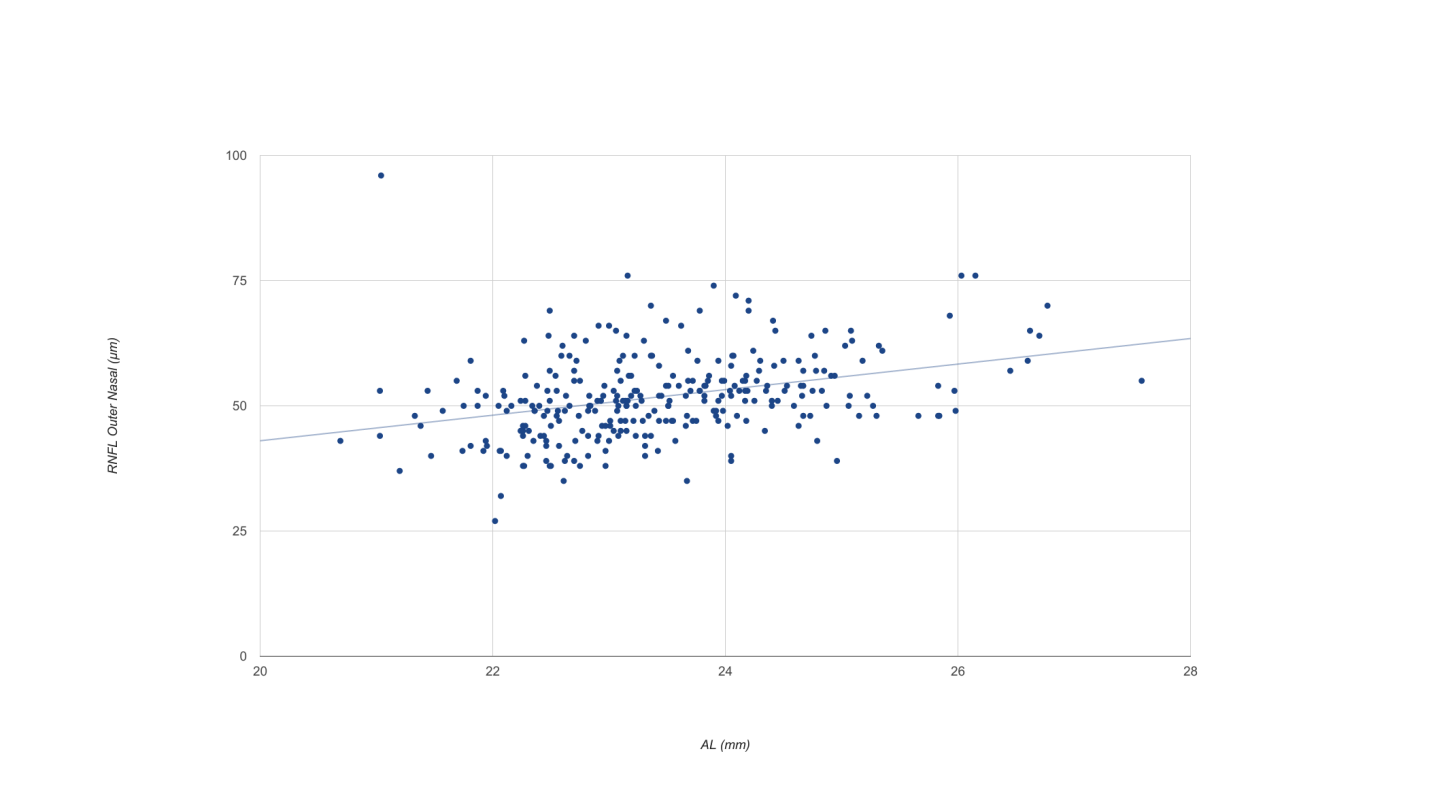
The mean nasal outer RNFL thickness in healthy Caucasian subjects as a function of axial length. AL = axial length.

The linear correlation analysis showed a positive correlation (r = 0.267) between the RNFL volume and the axial length (p < 0.001). The axial length had a significant effect on the RNFL thickness in every sector except for outer and inner temporal sectors. It had the greatest effect on the nasal outer sector (p < 0.001, r^2^ = 0.12), the equation for the regression line was y = 2.552 x AL—8.019 (Fig 1).

**Fig 2:**
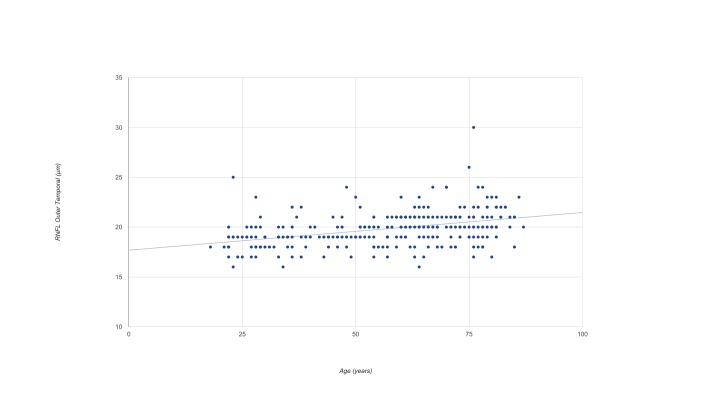
The mean temporal outer RNFL thickness in healthy Caucasian subjects as a function of age.

The linear correlation analysis showed a negative correlation between age and retinal volume (r = -0.351), between age and ganglion cell layer volume (r = -0.117), and age and inner plexiform layer volume (r = -0.321) (p<0.001, p = 0.043 and p<0.001 respectively). In Table 6, the results of the linear correlation analysis between macular thickness and age are shown. Age had the greatest effect on the RNFL temporal outer sector (p < 0.001, r^2^ = 0,163), the equation for the regression line was y = 0.038 x Age + 17,684 (Fig 2).

**Table 6:** Linear correlation analysis between macular thickness and age.

	RETINA		RNFL		GCL		IPL	
	r	p	r	p	r	p	r	p
**Central**	-0.026	0.661	0.035	0.545	-0.231	<0,001	-0.084	<0,001
**Nasal inner**	-0.329	<0,001	-0.111	0.057	-0.221	<0,001	-0.397	<0,001
**Nasal outer**	-0.399	<0,001	-0.152	0.009	-0.043	-0.043	-0.272	<0,001
**Superior inner**	-0.332	<0,001	-0.035	0.548	-0.121	0.038	-0.367	<0,001
**Superior outer**	-0.235	0,001	0.058	0.319	-0.058	0.322	-0.262	<0,001
**Temporal inner**	-0.243	<0,001	0.256	<0,001	-0.242	<0,001	-0.279	<0,001
**Temporal outer**	-0.217	<0,001	0.403	<0,001	-0.143	0.014	-0.144	0.013
**Inferior inner**	-0.327	<0,001	-0.125	0.032	-0.139	0.017	-0.382	<0,001
**Inferior outer**	-0.306	**<0,001**	-0.051	0.381	-0.037	0.524	-0.216	**<0,001**

The multivariate regression analysis adjusted by age showed that the retinal thickness was significantly higher (p<0.05) in men than in women, in every sector except for the temporal outer sector. The GCL thickness was significantly higher (p<0.05) in men in every sector except for the central sector. Statistically significant differences were found between both sexes only in the central, superior outer and temporal inner in the RNFL. Statistically significant differences were found only in the central, nasal inner, temporal outer and temporal inner sectors in the IPL.

## Discussion

The diagnosis of glaucoma on initial stages, before a significant functional loss is produced, is to this day a challenge and one of the greatest obstacles to clinical practice. The early detection of glaucoma enables an early treatment and a delay of the vision loss. Great efforts have been conducted to develop new imaging techniques, allowing us to detect glaucoma at an earlier stage. One of these methods is the spectral domain OCT. Its new structural parameters have been identified to measure the RNFL on the peripapillary area as well as the retinal thickness and the retinal inner layers thickness[7].

Normative databases for structural tests in glaucoma allow us to establish the reference values used to compare the patients with glaucoma or suspected glaucoma. Healthy subjects included on the normality databases should be carefully chosen. For the selection of patients, following the Realini et al. standards is recommended[6]. The population chosen should be representative of the population the test is going to be used for diagnostic purposes; the number of subjects should be sufficiently wide to characterize the reference population; variables such as age, axial length and race should be taken into account since they can affect the parameters; and, finally, the test must have inter- and intra-observer reproducibility.

In this study, the mean of the central sector was 278.2 +/- 21.12 um, these values are similar to the ones previously reported in the literature in Caucasian patients with the Spectralis OCT, 270.2 +/- 22.5 um[8]. The negative correlation between age and macular thickness is consistent with the information previously reported in the studies by Kanai et al. [9], Manassalorn et al.[10], and Appukuttan et al.[11], which showed a negative correlation with age in all the ETDRS sectors except for the central. This contrasts with other studies, where statistically significant differences with age were not found, as reported by Grover et al.[8], which could be caused by the significantly lower number of subjects (n = 50) in comparison to our study.

The present study demonstrated a significant decrease in GCL thickness and IPL thickness that increases with aging. This decrease is greater in the inner sectors than in the outer sectors. These results were similar to those reported in other OCT studies[11,12]. We found a positive correlation between age and RNFL thickness in the temporal sectors. Positive correlation between RNFL inner sectors thickness and age had been previously reported in the literature, Demirkaya et. al. reported a r = 0.216 (p = 0.019) in the RNFL inner sectors[12]. Regarding that the temporal sectors are the thinnest of the RNFL sectors, we postulated that the increase with age found in our study would be most probably caused by the internal limiting membrane being thicker in the older groups than in the younger groups[13].

The results of our study showed that the retinal thickness was significantly higher in men than in women. These data are consistent with previous studies, such as the studies by Appukuttan et al.[11] and Massin et al.[14], where they found that the mean retinal thickness was significantly higher in men in comparison to women.

The retinal thickness values were lower in the fovea and higher in the parafoveal area (inner circle), which is consistent with the normal retinal anatomy and with the data previously reported in the literature[11,15].

In our study, only Caucasian patients were included, so race was not taken into account for the analysis. Previous studies have showed statistically significant differences when comparing Caucasian patients to black patients[8,16]. These differences could be caused by the increase of pigment on the apical portion of the retinal pigment epithelium cells in black patients, which could mitigate the optical radiation incidence triggering lower retinal thickness values in comparison to white patients[17].

On the results of our study, a positive correlation between RNFL volume and axial length was found. This information has already been reported in the literature, the Akashi et al.[18] study found that the RNFL mean thickness on the macular area was thicker in myopic eyes than in non-myopic using OCT Cirrus.

The glaucomatous damage of the macula is common and it could be infradiagnosed with the campimetry strategies used in the usual clinical practice, such as the 24–2 test strategy[19]. Previous studies have proven that the thickness of the ganglion cell-inner plexiform layer is better as a parameter to detect glaucoma than the peripapillary RNFL in patients with central visual field affectation[20]. When comparing the RNFL of the peripapillary area with the RNFL of the macular area, as well as the GCL and the IPL, these last ones have a much lower inter-individual variability[21].

The measurements of thickness of the inner retinal layers could be useful not only for the early diagnosis of glaucoma but also as an instrument to measure the neuroprotective effect of the different therapeutic agents[20]. Since the central visual field is better conserved in advanced glaucoma, the CGL thickness in the macular area could be a parameter to measure the severity of terminal glaucoma when the peripapillary RNFL is no longer useful because of the floor effect[22, 23].

The thickness of the inner retinal layers is better as a parameter to detect the glaucomatous damage in the macula[19], while the peripapillary RNFL is better to detect the damage produced outside the macula, so continuing to develop new imaging methods to combine is necessary[20].

Our study was intended to establish the normal macular thickness values of the inner retinal layers, but new studies with healthy subjects and glaucoma patients are necessary to establish the diagnostic capacity of these values measured using Spectralis OCT.

Our study had some limitations. Only Caucasian patients were included, so our results cannot be replicated across other races. In our sample, there was a greater proportion of women than men and a higher proportion of subjects older than 51 years old which could have affected the end result. Since it is a transversal study, patients were not followed during the subsequent months to their inclusion in the study, consequently, it is possible that some of the patients have developed glaucoma or ocular hypertension during the months that followed.

## Supporting information

S1 Dataset Results of the macular inner layers thickness in healthy volunteers. (XLSX)
